# The Evolution of the Modern Hospital

**Published:** 1915-03-13

**Authors:** B. Burford Rawlings


### THE EVOLUTION OF THE MODERN HOSPITAL.*

### III.—The New Epoch in Equipment and Expense.

By B. BURFORD RAWLINGS.

EVEN the patients' lockers have shared the process of evolution. Not so many years ago they were rough cupboards, seldom if ever supervised as to their contents, in which a medley of incongruous and even malodorous articles was hoarded. To-day they are pleasing examples of the cabinetmaker's art, scrupulously clean because carefully inspected, and arranged with a view to the maximum of convenience. The bedsteads, if plain, as they ought to be, are no longer clumsy. In place of sagging canvas and the insanitary straw palliasse, with wool-filled mattress too often odorously offensive and seldom comfortable, they are fitted with removable wire-woven webbing, cleanly and supple, carrying a mattress stuffed with horse-hair; they have castors cased with rubber for easy and noiseless movement; the bed-linen is well kept, and the day quilts are of a quality and workmanship to invite the utmost of feminine appreciation. Down to the furthest detail of domestic equipment evidences of thoughtfulness are forthcoming, and flawless ward crockery and cutlery witness to the changes reached since the time when an unbroken knife was an exception and one polished unknown, when the forks were prongs of discoloured steel, when the teaboard was commonly furnished with cracked cups and handleless mugs, and the condiments of the cruet were represented by vinegar in a ginger-beer bottle.

The morning preparation of the ward is as serious a business as the dressing of a shop window, and although early entrance of the floor-scrubbers is not always pleasant to the patients and furniture polishing is disturbing, the final adornments are followed usually with interest and satisfaction. If it be true that sick folk are influenced for bodily good by mental processes, and that to provide bright and orderly surroundings is to promote recovery, the inmates of modern hospitals must possess advantages not only over those of their class treated in their own poor and crowded homes, but over many who incur none of the disabilities of poverty. Visitors from outside, when of the right sort, not impatient of needful restriction, prudent in their intercourse with patients, and capable of striking the right note in individual instances, are welcome trespassers upon routine, and furnish evidence of the gentle conditions which govern hospital domestic life to-day. Patients are now recognised as something more than merely passive recipients of service, given in the rough and without attempt at rectification. It is no longer demanded of them that they should take what is offered with their eyes shut, or that they must be indifferent to the promptings of elementary decency and refinement. Instead of exposure, as was at one time customary, to the view of the whole ward and even of visitors entering it, a patient is screened before receiving the attentions of the medical officer or the nurse. In the same way patients are saved the painful sight of fellow-sufferers *in extremis*, and the removal of the dead, at one time effected without regard to the feelings of the living, is now performed with reverence, and, as far as possible, with secrecy.

These are boons to a large majority of sufferers, to be rightly appraised only by those who can recall the keen distress caused to sensitive patients by wholly unnecessary publicity, displayed in occasional protest and in many silent but not less piteous appeals. The recreations provided for patients able to enjoy them are in themselves testimony to the kindly disposition of modern hospital service. To an inmate not ill enough to be indifferent, perhaps laid by with a sprained or broken limb, and in other respects sound, little could be more irksome than the unbroken blank of a long, unoccupied day passed in a ward. Besides music, for which therapeutical powers have been more than once claimed seriously, table games are supplied, and in not a few hospitals pleasant social intercourse is promoted, culminating in concerts and entertainments.

That the hospitals are now severally equipped with these and other invaluable domestic assets is matter for unalloyed satisfaction, but, like other good things, present results were not obtained without cost. The evolution of the modern hospital has been expensive. An increased outlay upon buildings and equipment has been accompanied by a serious enlargement of the cost of maintenance, brought about in part, no doubt, by the growth of the work, but largely by the conditions under which the work is carried on, the manifold requisitions of the medical staff, and the absence of effective means of controlling expenditure. Like private citizens, the hospitals must live up to the standard of their habitations. The substitution of buildings and appointments of the character now deemed necessary for those of more primitive pretensions is invariably followed by all-round additions to current expenses.

We seem to have reached this position, that while the hospitals have done much to set their houses in order, we have not succeeded, so far, notwithstanding the work of great and beneficent agencies established in aid, in securing an equilibrium of receipts with the expenditure as it is, while some costly items material to a perfect system of medical relief are not included. Yet real efficiency must ultimately depend upon that achievement. Time after time it has been shown how much easier is the task of providing new buildings or other improvements than of maintaining the establishment afterwards. The widespread interest and support which the hospitals ought to command is still wanting, and, notwithstanding all that has been written, they remain dependent upon the contributions of a very limited class of the community, and that not the richest.

With the entry of St. Bartholomew's, a short time ago, upon a career of building and begging, the last and only example of medical charity free of mendicancy disappeared.

* Articles I. and II. appeared on February 20 and March 6.

